# Factors Affecting Employee’s Retention: Integration of Situational Leadership With Social Exchange Theory

**DOI:** 10.3389/fpsyg.2022.872105

**Published:** 2022-07-11

**Authors:** Wei Xuecheng, Qaisar Iqbal, Bai Saina

**Affiliations:** ^1^School of Management, Universiti Sains Malaysia, George Town, Malaysia; ^2^Centre for China-India-Pakistan Studies, Sichuan University of Science and Engineering, Zigong, China; ^3^School of Chemistry and Environmental Science, Inner Mongolia Normal University, Hohhot, China

**Keywords:** compensation, staff retention, working environment, job satisfaction, sustainable leadership

## Abstract

Sketching on the Social Exchange Theory (SET), the present study aims to investigate the direct relationship between training and development, work environment, and job satisfaction with employee retention. The contingent role of transformational leadership was also analysed under the Situational Leadership Theory (SLT). Accordingly, we collected data from 287 employees of SMEs in northern China by employing a convenience sampling approach, exhibiting a response rate of 57.40 percent. The Partial Least Square-Structural Equation Modelling (PLS-SEM) analysis was then run to test the proposed hypotheses. The findings revealed a significant positive impact of training and development, work environment, and job satisfaction on employee retention. However, no moderating effect of transformational leadership was indicated on their direct relationship. This study has enriched the literature on employee retention and the leadership arena. To the best of the authors’ knowledge, there is no prior evidence concerning the study’s integrated relationship of the continuous variables. The implications and limitations were finally expressed at the end of this manuscript.

## Introduction

Employee retention is intricate in a competitive market, albeit vital for the long-term competitive advantage and organisational success and longevity (Das and Baruah, 2013; Arachchillage and Senevirathna, 2017; Kaur, 2017; Mahan et al., 2018; Paul and Vincent, 2018). The current COVID-19 situation has seen employee retention emerge as the core problem for organisations across the globe (Karatepe and Olugbade, 2017; Yousaf et al., 2019). Low employee retention results in various issues, that is, increased training and recruitment cost, insufficiently skilled employees, and disruption to organisational operations (A’yuninnisa and Saptoto, 2015; Ping et al., 2021). Due to these circumstances, small and medium enterprises (SMEs) view employee retention as highly complex and uncertain (Park et al., 2019; Tian et al., 2020).

In China, SMEs are the driving force of its social and economic development (Hadj, 2020; Hui, 2021), though they are presently finding it hard to operate at their full efficiency. This situation is exasperated by the COVID-19 pandemic, limited resources, low anti-risk capabilities, and diminished production scale (Zhanjie et al., 2017). These SMEs face bankruptcy and employee retention (Hui, 2021) due to adverse market conditions and economic uncertainty (Yu X. et al., 2019). China possesses approximately 770 million people in its workforce (Zhang and Chen, 2019), albeit maintaining the most significant global average turnover rate, that is, 18% (Friedman and Kuruvilla, 2015; Yu X. et al., 2019). Hence, practitioners and academicians continuously report the severity of employee turnover in China (Karatepe and Olugbade, 2017; Afsar et al., 2018).

High employee turnover weakens employees’ commitment and sets up negative perceptions of organisations (Hadj, 2020). Privately owned enterprises in China reported a 20% turnover rate, while state-invested enterprises and foreign-invested enterprises displayed 8% and 15%, respectively. Such staggering employee turnover is a pressing issue for Chinese SMEs as they grapple with managing employee retention (Zhang and Chen, 2019; Hu, 2021). Thus, practical managerial tools must be employed to alter employees’ behaviours (Choi and Peng, 2015). Given these points, it is imperative to investigate the underlying factors to enhance employee retention, considering the scarcity of research in China (Hom et al., 2017; Yousaf et al., 2019; Li et al., 2021).

Organisations are currently discussing varying strategies and practices to preserve their employees (Tanwar and Prasad, 2016; Bibi et al., 2018). Employee retention is a process through which employees are influenced to stay with their organisations for a longer period (Hom and Griffeth, 1995). Generally, employees are easy to retain, provided they see a good match with their employer (Umamaheswari and Krishnan, 2016). Extent literature concluded the significant role of various elements in relation to employee retention, such as intrinsic and extrinsic motivation factors (De Sousa Sabbagha et al., 2018), job promotion (Woodall et al., 2017), and bonus (Chinyio et al., 2018). Others include organisation commitment (Perreira et al., 2018), compensation (Colson and Satterfield, 2018), and knowledge sharing (Agarwal and Islam, 2015). This list can be extended to peer support, organisational culture, and work-life balance (Deshwal, 2015; Ombanda, 2015).

Notably, career development opportunities, benefits and rewards, and psychological factors are deemed vital for employee retention (Bibi et al., 2018; Lyman et al., 2020). Academicians and practitioners have developed a consensus about the crucial role of human resource management in developing this idea (Deshwal, 2015; Tian et al., 2020). Drawing on the basis of Social Exchange Theory (SET), this study aims to examine the direct effect of training and development, work environment, and job satisfaction on employee retention among China’s SMEs.

The leaders in an organisation commonly initiate change, execute, and interconnect with the desired results (Bass and Avolio, 1996; Bass and Riggio, 2006). In essence, employee retention is also not possible without effective leadership (Covella et al., 2017). In the last few years, numerous leadership styles have been examined concerning employee retention, such as laissez-faire, instrumental, transformational, and transactional leadership (Antonakis and House, 2014; Iqbal, 2016). Transformational leadership highly impacts employee commitment in contrast to transactional leadership (Deichmann and Stam, 2015). These leaders are highly concerned about real-time problems and establish new benchmarks, develop understanding, shape employees’ behaviours, and accomplish organisational objectives (Middleton et al., 2015; Tian et al., 2020; Iqbal et al., 2021a).

Transformational leadership comprises four dimensions, that is, individual consideration, intellectual stimulation, idealised influence, and inspirational motivation. The first dimension, individualised consideration, is the extent to which leaders understand and prioritise the team member’s needs. Meanwhile, intellectual stimulation is the extent leaders offer support and encourage employees to generate innovative ideas beneficial to delivering optimum performance. Moreover, inspirational motivation provides necessary support to the employees, enabling them to pursue organisational goals. The final dimension, idealised influence, encourages practical examples of a leader exhibiting innovative thinking, faith, pride, uprightness, interest, effective communication, and trust (Bass and Riggio, 2006).

Employees in China prefer leaders who exhibit transformational leadership attributes in the form of role models, non-use of abusive power, selflessness, and centring on employees’ well-being (Su et al., 2019). Hence, transformational leadership has become one of the most crucial roles in the organisational success of China’s SMEs (Lin and Sun, 2018). The current pandemic has resulted in economic uncertainty, environmental challenges, and the suitability of transformational leadership. Therefore, the Situational Leadership Theory (SLT) is applied to investigate its moderating role in the proposed relationship of training and development, work environment, and job satisfaction with employee retention.

Numerous contributions are made in this study concerning the theory and literature. First, the study developed the SET by revealing the direct impact of work environment, job satisfaction, training, and development on employee retention. Second, the insights on the conditional role of transformational leadership were elaborated in the context of SLT. The literature presented conflicting results and lacked clear explanations of the relationship’s nature of antecedents with employee retention (Abeysekera, 2007; Haines et al., 2010; Mangi et al., 2011; Ahmad et al., 2017). In this context, further studies are recommended to better comprehend the training and employee retention relationship (Bibi et al., 2018). Given these points, this study enriches the empirical evidence, specifically regarding the direct connection of training and development, work environment, and job satisfaction with employee retention. The final contribution included the literature on employee retention from the perspective of China’s SMEs.

## Literature Review

### Theoretical Background

The SET is widely applied to unravel the employer–employee relationship, especially in the employee turnover and retention literature (Coyle-Shapiro and Conway, 2005; Gopalan et al., 2020). According to this theory, a person, who benefits from someone, feels obligated to repay that person through positive behaviours and devotion. Furthermore, this theory postulates that employees deliver their optimum performance upon achieving support and perceiving value from their employers (Eisenberger et al., 2001). Hence, the theory is used to investigate the employees’ behaviour, enabling organisations to enforce certain HRM practices and igniting unique social exchange relationships.

From the SET perspective, employee retention can be induced by training and development offered by employers, which facilitate mutual benefits and create reciprocated obligations. This phenomenon occurs because individuals and organisations are involved in exchange relationships (Raihan, 2012). Employees perceive responsibility to repay their employers upon providing a conducive working environment. This repayment may derive in the form of increased loyalty, commitment, and stay for a long time (Settoon et al., 1996). Simultaneously, their job satisfaction and proper behavioural responses will increase, owing to the perception of fulfilling emotional needs (Iqbal and Hasnah, 2016; Latorre et al., 2016; Iqbal et al., 2017; Rubel et al., 2021) and improving employee retention (Rubel et al., 2021). Therefore, the current study posits that job satisfaction and work environment followed by training and development are positively related to employee retention.

A specific type of leadership is required to tackle the distinct needs and current challenges of a particular environment. Hence, according to the SLT, a single leadership style is insufficient for every situation (Hersey and Blanchard, 1969). In this case, effective leadership, that is, transformational leadership, emerges as a promising idea and is applicable across diverse fields. This concept facilitates the concept of adapting to varying circumstances and work environments (Hersey and Blanchard, 1969). In the context of employee retention, an environment must be structured where they feel empowered, valued, and connected to their employers (Ohunakin et al., 2019; Frye et al., 2020); thus, this leadership style is consistent with these requirements (Kim and Park, 2020). Consequently, this leadership style enhances the employees’ capability and reshapes the organisational image in the marketplace (Mwita and Tefurukwa, 2018; Singh et al., 2020). Drawing on the SLT, the current research claims the moderating role of transformational leadership on the relationship of training and development, work environment, and job satisfaction with employee retention.

### Hypotheses Development

#### Training and Development With Employee Retention

Training and development is the degree to which training within the organisation is offered to the employees to foster their skills (Delery and Doty, 1996). As an overarching HRM practice, it is often considered a broad collection of activities that refer to continual learning and developing general job and career-related skills (Boon et al., 2011). Furthermore, training is the fundamental source of competitive advantage and employee retention (Umamaheswari and Krishnan, 2016; Yamin, 2019). Training and development intensify the social exchange relationship between the employee and their employer (Dysvik and Kuvaas, 2008), offering employees valuable abilities, skills, and knowledge (Fletcher et al., 2018). This idea elicits obligations within employees to repay the organisation (Koster et al., 2011).

Training and development programmes deal with the employees’ skills and competencies, enabling them to positively respond to various challenges the organisations face (Rhee et al., 2014). Moreover, positive dispositions of employee growth can be achieved *via* motivation and modifying their skills or attitude toward organisational effectiveness (Gope et al., 2018; Yamin, 2019; Khan et al., 2021). These skills and competencies are vital for their managerial positions and professional growth (Schuler and Tarique, 2012; Ambrosius, 2018). Past studies have found a positive relationship between training and development with commitment (Ahmad et al., 2017), employee performance (Sinha et al., 2010), and job satisfaction (Bibi et al., 2018). Others include employee retention (Lee, 2005), employee commitment (Ahmad et al., 2017), and employees’ intentions to stay (Chew and Chan, 2008). Therefore, the hypothesis H1 is postulated as follows:

*H1: Training and development significantly and positively influence employee retention.*

#### Work Environment and Employee Retention

The working environment concerns the availability of a conducive workplace (Edgar and Geare, 2005) and is defined as the degree to which employees consider the workplace physically safe. Employees can share their views on their surroundings with their mutual consideration with organisations by assessing the environment (Lewin et al., 1939; Li et al., 2022). Some examples of work environment indicators include supervisor support (Stirpe and Zárraga-Oberty, 2017), physical working conditions (Richards et al., 1994), social worker support (Haggins, 2011), and helping behaviours during decision-making (Subramaniam and Mia, 2001). Notably, a study found a significant relationship between work environment and employee retention (Al-Hamdan et al., 2017).

Organisational rules and regulations encompass the work environment, affecting employee retention (Yam et al., 2018). Consequently, an exceptional working environment increases trust among employees, which is useful for employee retention (Candela et al., 2015; Ede and Rantakeisu, 2015). The positive energy motivates employees to accomplish their professional goals effectively, enhancing their commitment to the organisation (Mangi et al., 2011; Umamaheswari and Krishnan, 2016). The perception of the working environment can either positively or negatively impact specific employees’ job outcomes, such as commitment, participation, and intention to stay (Gunaseelan and Ollukkaran, 2012). Given these points, the discussion above drives the development of hypothesis H2:

*H2: Work environment significantly and positively influences employee retention.*

### Job Satisfaction and Employee Retention

Job satisfaction concerns employees’ evaluations of their jobs based on perceptions by comparing their actual job outcomes with desired ones (Schleicher et al., 2011). The concept is defined as a positive state where employees share their feelings about their job (Locke, 1976), ranging from moderate- to low-level satisfaction (Locke, 1976; Quigley et al., 2007). Furthermore, the idea is considered a causal factor that promotes intentions to stay with the organisation because it is a pleasant psychological state. In this case, individuals perceive content with their work (Fletcher et al., 2018). Therefore, job satisfaction fosters the social exchange between employers and employees, where satisfied employees exemplify positive experiences. This experience is supported by the social exchanges between the employee and the organisation, reinforcing their intentions to stay with the organisation (Koster et al., 2011).

Employees from varying organisations, industries, and geographical locations exhibit different levels of job satisfaction (AbuAlRub et al., 2009). However, job satisfaction positively affects employees’ intentions to stay irrespective of industries and region (AbuAlRub et al., 2009). For instance, a multi-level study found that job satisfaction is positively related to the employees’ intentions to stay in the united kingdom (UK) (Fletcher et al., 2018). Moreover, meta-analytic evidence has demonstrated that those who are satisfied at work are more likely to retain their employment (Fishbein and Ajzen, 2011); therefore, the hypothesis H3 is proposed on the basis of SET as follows:

*H3: Job satisfaction significantly and positively influences employee retention.*

### Moderating Role of Transformational Leadership

Transformational leadership is considered highly effective in driving employee retention (Kossivi et al., 2016; Amankwaa et al., 2019), in which the leaders initiate, execute, and interconnect change with the desired outcomes (Bass and Riggio, 2006). This form of leadership influences employees by altering their perceptions, views, ambitions, and moral standards (Bass and Avolio, 1996). Transformational leaders also offer an ideal approach to employees and exhibit the attributes of significant faith, effective communication, uprightness, trust, and innovative thinking (Tian et al., 2020). These leaders promote intellectual stimulation, idealised influence and inspirational motivation, and individualised consideration. In addition, leaders can facilitate employees by understanding their issues and creating a psychologically safe environment through individualised consideration (Iqbal et al., 2021b).

Transformational leaders are viewed as role models and counsellors who encourage them to participate in organisational activities. Supervisor support, training, information accessibility, and counselling lead to higher employee retention (Ooi et al., 2021) and higher employability (Matsuo, 2021). Furthermore, healthy communication positively influences the work environment (Denton, 2011) *via* sharing of innovative ideas and intellectual stimulation, an indicator of open communication. This approach culminates in improved work that enhances talent management (Perlow and Kelly, 2014). Moreover, open communication and improving resource management in employee allocation ease employees’ adaptation to new work environments (Castrogiovanni et al., 2011).

Organisational support in the form of supervisor support, rewards, and favourable working conditions are vital to stimulating organisation-related outcomes, that is, reduced withdrawal behaviours and commitment (Rhoades and Eisenberger, 2002; Gillet et al., 2022). Supervisor support is critical to replenish employee physical and psychological resources to increase their retention probability (Kalliath and Kalliath, 2014). Furthermore, transformational leaders affect employees’ behaviour resulting in high employee retention (Sow et al., 2016). Leaders can develop a reward system within their organisation which is highly effective in retaining employees (Adekanbi, 2016). They must also ensure sufficient resources to employees following the organisational goals. Moreover, shared vision is directly related to the employees’ engagement (Boyatzis et al., 2017) and continuous improvement (Fardazar et al., 2015; Iqbal and Piwowar-Sulej, 2022). This leadership style presents a shared vision and elaborates the underlying reasons, enhancing their participation and involvement in decision-making. Previous studies found that transformational leadership negatively impacts employees’ turnover intention (Maaitah, 2018) but positively affects their knowledge base (Fletcher et al., 2018). For instance, a positive impact of various factors on employee retention, that is, idealised influence, inspirational motivation, individualised consideration, and intellectual stimulation (Jiang et al., 2017). Therefore, the following hypotheses, H4, H5, and H6, are proposed:

*H4: Transformational leadership significantly moderates the training and development—employee intentions relationship.*
*H5: Transformational leadership significantly moderates the work environment—employee intentions relationship.*
*H6: Transformational leadership significantly moderates the job satisfaction—employee intentions relationship.*

**FIGURE 1:**
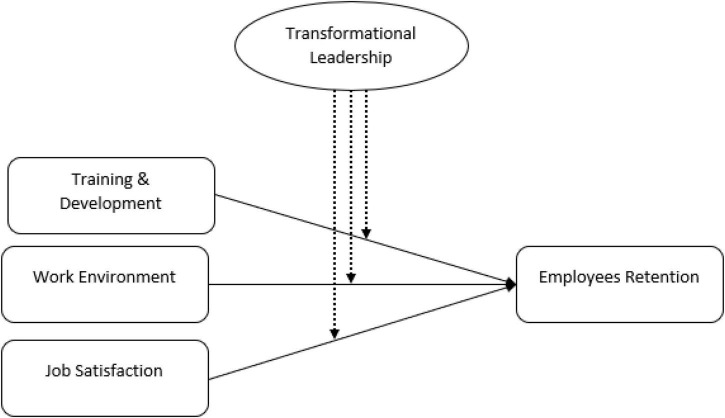
Research framework.

With reference to the above discussion, the hypothesized model is drawn as shown in the below Figure 1.

## Research Methodology

### Context, Sample, and Data Collection

In China, organisations face fierce pressure to retain employees due to the shortage of skilled and talented employees (Fu et al., 2020). Statistically, 44% of the top management in organisations operating in China view this issue as a critical barrier to employee retention (Mashiah, 2021). China’s manufacturing makes up two-thirds of SMEs (Zhu et al., 2012; Iqbal et al., 2021c; Xuecheng et al., 2022), where most operate in the northern region. Therefore, this study focuses on the SME employees in this region. Moreover, this study requires a minimum of 185 responses based on the sample-item ratio (Hatcher and O’Rourke, 2013). Online survey forms were structured to collect data, comprising six sections that measure various factors. The factors include training and development, work environment, job satisfaction, employee retention, transformational leadership, and respondents’ demographics.

We adopted a convenience sampling approach for data collection, considering China’s time and financial constraints and current COVID-19 restrictions. The present study is cross-sectional in design, where data are collected at a specific time from the employees. The online survey link was disseminated *via* 500 email addresses with the assistance of the human resource department. Accordingly, 287 responses were received, which is sufficient, indicating a response rate of 57.40%. Furthermore, a gentle reminder was included during data collection to increase the number of responses. In this study, we also marked it mandatory to check against each item in the online survey form, ensuring no missing values in the dataset.

### Measurement of Variables

We adopted measurement scales of four continuous variables in the current study. Previous studies have reported reduced quality and high cognitive ability required to collect data using a high Likert scale (Cummins and Gullone, 2000; Iqbal et al., 2020). Therefore, a 5-point Likert scale was employed, ranging from 1 = strongly disagree to 5 = strongly agree. Delery and Doty (1996) have defined training and development as the degree to which organizations offer training to employees to foster their skills. We adopted the 4-item scale from Delery and Doty’s (1996) study to measure training and development. For example, one item is “We receive formal development training which increases our promotion chances within the organisation.” Similarly, Bibi et al. (2018) used an identical scale in the context of Pakistan and found it highly reliable (α = 0.918). In this study, the Cronbach’s alpha value of this scale is 0.719.

The work environment is defined as the degree to which employees perceive the availability of a safe and conducive workplace (Edgar and Geare, 2005). Subsequently, 4-measurement items were adopted from Bibi et al.’s (2018) study to assess the work environment. For instance, one item is “We always feel safe working here in this environment.” In the current study, the Cronbach’s alpha value of this 4-item scale is 0.928. Meanwhile, job satisfaction is defined as the pleasurable emotional state emerging from the job appraisal as facilitating the accomplishment of one’s job values (Locke, 1976; Zhang M. M. et al., 2016). In this case, we adopted a 3-item scale from Cammann et al.’s (1979) study to measure job satisfaction. An example of this item is “In general, I like working here.” This scale was utilised by Zhang L. et al. (2016), who found it highly reliable (alpha = 0.870). Accordingly, the Cronbach’s alpha value in this study for the 3-item scale is 0.921.

Transformational leadership is defined as those who have idealised influence, intellectual stimulation, inspirational motivation, and individualised consideration. We adopted 20 items of the Multifactor Leadership Questionnaire (MLQ) to analyse transformational leadership. This analysis was related to the four items, namely intellectual stimulation, idealised influence, inspirational motivation, and individualised consideration. A sample of the item is “my leaders give me tasks with enthusiasm.” A previous study (Ohunakin et al., 2019) indicates high reliability where Cronbach’s alpha values of its four dimensions were between 0.88 and 0.92. In this study, the Cronbach’s alpha values are in the range of 0.875–0.918.

Employee retention is defined as the effort by an organisation to keep desirable employees to fulfil business objectives (Frank et al., 2004; Govaerts et al., 2011). We adopted six items (Govaerts et al., 2011) to measure employee retention, for example, “I love working for this company.” The present study’s measurement scale was highly reliable, that is, Cronbach’s alpha = 0.794, aligning with Khalid and Nawab’s (2018) findings.

### Analytical Approach

In this study, the research framework is complex due to its prediction-oriented feature and the presence of independent variables, dependent variables, and moderators. Therefore, partial least squares-structural equation modelling (PLS-SEM) was selected following Hair et al. (2020). This approach is considered a proper analytical strategy compared to covariance-based structural equation modelling (CB-SEM) (Ringle et al., 2020). The technique revolves around the assessment of the measurement model and structural model, though it requires prior evaluation of the former measurement.

## Results of the Study

### Data Screening

Before analysis, data screening must be conducted, which concerns missing values, outliers, data normality, and common method bias. We ensured the absence of missing values in the current dataset by marking mandatory against each measurement item in the online survey form. Moreover, univariate outliers and multivariate outliers were investigated through Z-score and the Mahalanobis distance test. Accordingly, three responses were removed in the univariate outlier because of the Z-score values greater than 3.29 (Tabachnick and Fidell, 2007). Meanwhile, the Mahalanobis distance test revealed that the *P*-value of 15 cases is less than 0.001, a clear indicator of multivariate outliers; therefore, the 15 responses were excluded from the dataset.

The normality was assessed based on the skewness and kurtosis values, and in this study, the skewness values of all continuous variables extend from −1.307 to 1.531, which are within ± 3. However, the kurtosis values fall out of the ± 3 range (DeCarlo, 1997), and thus, the data are not univariate normal. The Mardia’s coefficient skewness 0.005 and kurtosis values (β=172.761,ρ < 0.005) confirmed the multivariate normality. Nevertheless, the PLS-SEM does not require data normality; hence, non-normal distribution is not an issue. Next, we applied Harman’s single factor test and the correlation matrix procedure to examine the common method bias. Harman’s single factor test revealed that a single factor only counts for 39.41% < 0.50% of the total variance (Podsakoff et al., 2012), confirming no issues with method bias. The correlation matrix exposed that no single correlation between continuous variables is greater than 0.90 (Bagozzi et al., 1991); thus, the current study is free from common method bias. We have also examined the model fit of hypothesized model based on two-index strategy recommended by Hu and Bentler (1999). In the present study, hypothesized model was found fit based on comparative fit index (CFI) (0.96 > 0.95) and standardised root mean square residual (SRMR) (0.049 < 0.09) (Hu and Bentler, 1999), as compared to alternative models.

### Frequency Analysis

The present study is dominated by male participants (*n* = 176, 61.32%), where most participants (*n* = 109, 37.98%) fall between the ages of 25 and 35, followed by 85 between 36 and 45. The most significant number of participants (*n* = 144, 50.17%) in current research possessed a bachelor’s degree and nine participants (3.14%) with PhDs. Finally, more than 50% of the participants (*n* = 153) acquired 5–10 years of working experience, while nine participants with more than 20 years of professional experience.

### Descriptive Analysis

In this study, the mean values of training and development (*M* = 4.145), work environment (*M* = 4.334), job satisfaction (*M* = 4.322), and employees’ retention (*M* = 4.167) were found significant. Notably, job satisfaction and employee retention values were higher than those reported by a previous study among employees of multi-national enterprises (MNEs) in China (Zhang M. M. et al., 2016). Regarding four dimensions of transformational leadership, idealised influence (*M* = 4.132) has the highest mean value. This result is followed by inspirational motivation (*M* = 4.121), individualised consideration (*M* = 3.973), and intellectual stimulation (*M* = 3.670). Moreover, the current descriptive analysis revealed that participants seek more idealised influence than other dimensions of transformational leadership in Chinese SMEs. In other words, a leader’s ability to exhibit high morality, ethics, and personality enables the realisation of high performance within organisations.

### Measurement Model Analysis

The measurement model analysis examines the construct reliability and validity, in which the former is assessed with reference to its indicator and internal reliability. This study revealed that all indicator loadings are more significant than 0.50 and less than 0.944, which are deemed acceptable. An item has sufficient indicator reliability provided its factor loading is more significant than 0.50 (Chin, 1998). Moreover, we examined the construct reliability based on Cronbach’s alpha and composite reliability values. A construct has acceptable reliability, provided its Cronbach’s alpha or composite reliability value is greater than 0.70 (Sarstedt et al., 2019).

Table 1 shows that the Cronbach’s alpha values of various items are greater than 0.70. These items comprise training and development (0.719), work environment (0.928), job satisfaction (0.921), employees’ retention (0.794), and individualised consideration (0.889). Other items include idealised influence (0.918), inspirational motivation (0.875), intellectual stimulation (0.882), and transformational leadership (0.885). Similarly, the composite reliability values of these variables are greater than 0.70 (see Table 1). Hence, it is evident that all the continuous variables exhibit acceptable construct reliability.

**TABLE 1:** Factor loadings, reliability, AVE, and mean values.

Construct	Item	Loading	Cronbach’s alpha	Composite reliability	Average Variance Extracted (AVE)	Mean
Training and development	TD1	0.740	0.719	0.825	0.541	4.145
	TD2	0.720				
	TD3	0.730				
	TD4	0.752				
Work environment	WE1	0.864	0.928	0.939	0.823	4.334
	WE2	0.926				
	WE3	0.940				
	WE4	0.897				
Job satisfaction	JS1	0.929	0.921	0.930	0.864	4.322
	JS2	0.944				
	JS3	0.915				
Employees retention	ER1	0.777	0.794	0.890	0.576	4.167
	ER2	0.732				
	ER3	0.827				
	ER4	0.733				
	ER5	0.813				
	ER6	0.658				
Individualized Consideration (IC)	IC1	0.931	0.889	0.905	0.706	3.973
	IC2	0.857				
	IC3	0.725				
	IC4	0.836				
Idealized Influence (II)	II1	0.772	0.918	0.937	0.712	4.132
	II2	0.803				
	II3	0.888				
	II4	0.850				
	II5	0.873				
	II6	0.869				
Inspirational Motivation (IM)	IM1	0.862	0.875	0.910	0.669	4.121
	IM2	0.845				
	IM3	0.845				
	IM4	0.731				
	IM5	0.798				
Intellectual Stimulation (IS)	IS1	0.920	0.882	0.916	0.692	3.670
	IS2	0.887				
	IS3	0.915				
	IS4	0.523				
	IS5	0.846				
Transformational leadership	IC	0.896	0.885	0.891	0.677	3.982
	II	0.923				
	IS	0.853				
	IM	0.572				

*Means multiplication/interaction of two variables.

Construct validity is formulated on the convergent and discriminant validity, where a construct has sufficient acceptable convergent validity provided its factor loadings are greater than 0.70. Furthermore, its average variance extracted (AVE) must be higher than 0.50 (Hair et al., 2020). The items of all continuous variables must possess factor loadings greater than 0.70 (see Table 1). The AVE values of multiple items revealed values higher than 0.50, that is, training and development (0.541), work environment (0.823), and job satisfaction (0.864). Others include employee retention (0.576), individualised consideration (0.706), idealised influence (0.712), inspirational motivation (0.669), intellectual stimulation (0.692), and transformational leadership (0.677) (see Table 1).

Based on the above results, all continuous variables possess acceptable convergent validity. We employed Fornell–Larcker Criterion to examine the discriminant validity of the variables. This method confirms the discriminant validity of a variable provided that the square root of its AVE is greater than its inter-constructs correlation values (Henseler et al., 2009). Table 2 indicates that the square root of AVE of all variables is greater than their inter-constructs correlation values; hence, these variables exhibit acceptable discriminant validity.

**TABLE 2:** Fornell–Larcker criterion.

Constructs	1	2	3	4	5
Employee retention	**0.759**				
Job satisfaction	0.655	**0.930**			
Training and development	0.743	0.679	**0.735**		
Transformational leadership	0.675	0.445	0.533	**0.823**	
Work environment	0.625	0.902	0.723	0.392	**0.907**

*The bold value stands for the square root of the AVE value of respective variable.*

### Structured Model Analysis

The structural model analysis revealed that training and development significantly influence employee retention (β=0.824,ρ < 0.05) (Table 3). In essence, one unit change in training and development brings 82.40% variations in employee retention, and thus hypothesis H1 is supported. Notably, the findings indicated the significant positive impact of the work environment on employee retention (β=0.274,ρ < 0.05), supporting hypothesis H2. Meanwhile, job satisfaction significantly influences employee retention (β=0.824,ρ < 0.05) (see Table 3). In other words, there is a 20% change in employee retention among SME employees in China for one unit change in job satisfaction, supporting hypothesis H3.

**TABLE 3:** Hypotheses testing.

Hypothesis	β	S.D	*T* value	*P*-values	LLCI	ULCI
Training and development → Employee retention	0.824	0.071	11.615	0.000	0.685	0.963
Work environment → Employee retention	0.274	0.087	3.138	0.002	0.103	0.445
Job satisfaction → Employee retention	0.202	0.091	2.219	0.027	0.024	0.380
Job satisfaction * Transformational leadership → Employee retention	0.078	0.101	0.775	0.439	−0.120	0.276
Training and development * Transformational leadership → Employee retention	−0.081	0.068	1.193	0.233	−0.214	0.052
Work environment * Transformational leadership → Employee retention	0.021	0.114	0.182	0.856	−0.202	0.244

The effect of its interaction terms with training and development, work environment, and job satisfaction were estimated. This approach was conducted to examine the moderating effect of transformational leadership. In this case, the interaction term of transformational leadership with various dimensions does not significantly influence employee retention in SMEs in China. The dimensions include training and development (β = −0.081,ρ=0.233 > 0.05), work environment (β=0.021,ρ=0.856 > 0.05), and job satisfaction (β=0.078,ρ=0.101 > 0.05) (see Table 3). Therefore, moderation hypotheses H4, H5, and H6 are rejected.

## Discussion

The current study examined an essential topic in organisational behaviour: what factors are vital to foster employee retention in SMEs? Accordingly, a research framework was proposed and empirically tested based on the SET to analyse the impact of multiple dimensions (training and development, work environment, and job satisfaction) on employee retention. Similarly, the conditional effect of transformational leadership was analysed based on this relationship. Current findings confirmed the positive connection among the three dimensions of employee retention. However, the contingent role of transformational leadership was not supported by the proposed relationship. Only three direct hypotheses are supported in this study, and the findings are elaborated below.

The SET was applied in this study to propose the positive relationship of the three dimensions with employee retention. The data analysis purported the significant positive relations of training and development with employee retention among SME employees in China; therefore, supporting hypothesis H1. This finding aligned with previous studies (Zheng, 2009; Umamaheswari and Krishnan, 2014; Bibi et al., 2018). Past studies reported a positive impact of training and development on employee retention in Pakistan’s universities (Bibi et al., 2018) and Indian ceramic industries (Umamaheswari and Krishnan, 2014). A similar observation can be found in the hotel industry of Bangladesh (Rubel et al., 2021) and multinational enterprises in Asia (Zheng, 2009).

A study among millennial employees in Bangladesh concluded a significant positive effect of green training and development on employee retention (Islam et al., 2022). Meanwhile, training and development reported a significant indirect impact on employee retention through ethical climate (Yamin, 2019) and employee engagement (Fletcher et al., 2018). Another study reported a negative link between the practices of perceived human resource management and turnover intention among SME employees (Reese et al., 2009). Deng (2018) similarly claimed that family business retains migrant workers by fostering training and development programmes. Therefore, organisations must carefully design and implement these programmes to increase employee retention.

The current research indicated a positive relationship between work environment and employee retention. The results supported this proposition, resulting in the acceptance of hypothesis H2, aligning with previous findings (Pek-Greer and Wallace, 2017; Frye et al., 2020; Wu et al., 2020). Other studies echoed the current study’s results. For instance, the work environment was positively related to employee retention among generation Y (Frye et al., 2020). Meanwhile, a qualitative study in Singapore suggested that a supportive work environment strongly predicts employee retention in its education sector (Pek-Greer and Wallace, 2017). In China, the work environment indirectly influences employee turnover through workplace violence (Wu et al., 2020) and India’s organisational engagement (Kundu and Lata, 2017). Other studies supported the positive impact of the work environment on the employee turnover intention in China’s health sector (Wan et al., 2018; Wu et al., 2020).

This study proved the significant positive effect of job satisfaction on employee retention based on hypothesis H3, leading to its acceptance, parallel to previous findings (Tanwar and Prasad, 2016; Frye et al., 2020). A study found that job satisfaction positively affects employee retention in hospitality (Frye et al., 2020). Meanwhile, a qualitative study among IT industry employees showed that employer branding vis-à-vis job satisfaction strongly determines employee retention (Tanwar and Prasad, 2016). On a similar note, Liu et al. (2010) confirmed that job satisfaction is a strong predictor of employee retention in China’s health centres. Zhang M. M. et al. (2016) similarly supported this positive relationship among Chinese employees working for multinational enterprises. In the service industry, the aforementioned three dimensions are viewed as vital factors in promoting employee retention (Mohanty and Mohanty, 2016).

The present study introduced the moderating effect of transformational leadership on the relationship of the three dimensions with employee retention. A transformational leader is anticipated to significantly moderate the training and development-employee retention relationship, though the findings do not support this proposition. Hence, H4 is rejected. In this context, there is no prior study on leadership as a moderating variable on the link between training and development with employee retention. However, a study suggested a green creativity climate as the potential moderator of the green practices-employee retention link among millennial employees (Islam et al., 2022).

The moderating role of transformational leadership on the work environment-employee retention relationship was not supported. This result is a clear indicator of the rejection of hypothesis H5. Similarly, there is no study regarding leadership as a contingent variable on the work environment-employee retention association. Thus, the current study offers strong empirical contributions to the field of training and development followed by the work environment. Meanwhile, job satisfaction possesses substantial weightage in employee retention, and this relationship relies on the organisational climate (Sips et al., 2015). The current research established the conditional effect of transformational leadership on the job satisfaction-employee retention link.

However, the present findings do not support hypothesis H6, contradicting Sips et al.’s (2015) findings. The underlying reasons are due to the leaders’ direct role in developing organisational climate rather than their immediate effect on job satisfaction. Moreover, another study revealed that servant leaders indirectly influence employee retention through job satisfaction (Hassan et al., 2021). In Nigeria, a significant positive effect of various elements was found on employee retention in universities. These elements include idealised influence, inspirational motivation, intellectual stimulation, and individualised consideration (Ohunakin et al., 2019).

## Conclusion

The current study aimed to investigate the direct effect of factors such as job satisfaction, working environment, and training and development on employee retention in China. This study also intended to examine the moderating role of transformational leadership in the relationship between job satisfaction, working environment, and training and development on employee retention based on cross-sectional data collected from employees in manufacturing SMEs in China. The current empirical findings confirm that all three factors such as training and development, job satisfaction, and the working environment significantly influence employee retention. Among these three variables, training and development have the highest positive effect on employee retention. Yet, present statistical findings do not support the moderating effect of transformational leadership on the relationship of job satisfaction, working environment, and training and development with employee retention.

### Theoretical Implications

The present study significantly contributed to the extension of the theory. First, the literature is enriched by offering empirical support on the integrated understanding of the three dimensions and employee retention. Furthermore, extant literature provided contradicting findings on the relationship between the three dimensions. However, the role of transformational leadership as the conditional variable in their relationship is still missing. Second, this study augments the SET by examining the effect of training and development, work environment, and job satisfaction on employee retention. Third, the SLT is extended by providing evidence on the non-significant moderating role of transformational leadership, specifically the correlation of the three dimensions on employee retention.

### Practical Implications

The current research offered several recommendations for practitioners and policymakers. Extant literature claims that employee retention is a significant challenge for SME employees in China. In this case, the current work emphasises the three dimensions critical to enhancing employee retention within China’s SMEs. The present findings found that training and development is the most crucial element which increases employee retention within organisations. Chinese SMEs may, therefore, plan their training and development programme more effectively based on their employee’s needs.

China’s SMEs should design training and development programmes that parallel their employees’ career growth. However, previous findings focused on offering training programmes related to company-specific skills rather than general skills. Accordingly, general skills enable employees to effectuate professional opportunities outside the parent organisations. Therefore, these SMEs must evaluate their programmes and perform changes concerning their company-specific skills. Equally important, the top management should employ specific strategies to foster a conducive workplace to improve the work environment and cope with employees’ burnout. Such strategies may include special counselling sessions for stress-induced employees and enforcing flexible work schedules.

The management should centre on developing the work environment, ensuring satisfied employees, establishing open communication, and fostering ideas while offering peer support. Moreover, practitioners should work on the proper fund allocation to develop a positive work environment. The current study reported a significant positive effect of job satisfaction on employee retention. Hence, the human resource and departmental managers are advised to make incremental changes, spurring employee satisfaction, that is, providing a fair salary, rewards, and incentives to their employees. Nevertheless, the moderating effect of transformational leadership does not appear significant in retaining employees for SME management in China.

Based on the above, it is proven that transformational leaders are highly effective in designing an empowered and meaningful work environment. For instance, this form of leadership offers individualised consideration and idealised influence, stimulates intellectually, and motivates inspiration. Thus, practitioners and managers should evaluate the development of leadership. Specifically, in China’s SMEs, policymakers, and managers must critically assess the leadership development programmes in their organisations.

### Limitations

The current study possesses several limitations despite the significant theoretical and practical contributions. First, this study was conducted in the northern region of China, hence, increasing the generalisation in the context of China. Moreover, the sole focus on China SMEs may not provide comprehensive information on other emerging and developed economies. Therefore, future studies can enrich the quantitative findings by assessing other developing countries, such as Pakistan and India. The survey form was distributed through the human resource management department, decreasing the chance of randomly distributing the survey form to employees. This situation will hinder the generalisation issue further, especially in a broader population.

Second, this study subjectively measured all continuous variables, and such measurement might influence the presence of common method bias. Practitioners and academicians face extreme difficulty in collecting objective data from organisations. Thus, future endeavours could overcome these limitations by adopting improved research design and employing a qualitative approach to unravel the causal relationship. Third, a direct connection was found between the three dimensions with employee retention. The upcoming research must then investigate the potential mechanisms of these relations. Finally, the current study provided multiple shreds of evidence about the non-significant moderating role of transformational leadership; thus, further examinations must be made on the conditional part of other leadership styles, such as sustainable and servant leadership.

## Data Availability Statement

The original contributions presented in this study are included in the article/supplementary material, further inquiries can be directed to the corresponding author.

## Author Contributions

All authors listed have made a substantial, direct, and intellectual contribution to the work, and approved it for publication.

## Conflict of Interest

The authors declare that the research was conducted in the absence of any commercial or financial relationships that could be construed as a potential conflict of interest.

## Publisher’s Note

All claims expressed in this article are solely those of the authors and do not necessarily represent those of their affiliated organizations, or those of the publisher, the editors and the reviewers. Any product that may be evaluated in this article, or claim that may be made by its manufacturer, is not guaranteed or endorsed by the publisher.
